# Patient‐specific psychological characteristics and personality structure affect functional outcomes after surgical stabilization of acute and chronic acromioclavicular joint injuries

**DOI:** 10.1002/ksa.70349

**Published:** 2026-02-24

**Authors:** Lukas Nawid Muench, Ali Can Gunenc, Marco‐Christopher Rupp, Lisa Rahn, Frank Martetschläger, Armin Runer, Lucca Lacheta, Sebastian Siebenlist, Bastian Scheiderer

**Affiliations:** ^1^ Department of Sports Orthopaedics TUM University Hospital Munich Germany; ^2^ ATOS Clinic Munich Germany

**Keywords:** AC joint injury, acromioclavicular joint, arthroscopically assisted AC joint stabilization, personality structure, psychological factors

## Abstract

**Purpose:**

The purpose of the study was to evaluate the impact of psychological factors on functional outcomes of patients undergoing isolated acromioclavicular joint (ACJ) stabilization for acute and chronic ACJ injuries.

**Methods:**

Patients who underwent ACJ stabilization between January 2015 and December 2021 and had a minimum follow‐up of 2 years were analysed. Functional outcome measures included Constant–Murley (CM), American Shoulder and Elbow Surgeons (ASES), Nottingham Clavicle (NC) scores and the Visual Analogue Scale (VAS) for pain. Patient‐specific psychological characteristics and personality structure were evaluated using the Tampa Scale of Kinesiophobia (TSK), General Self‐Efficacy Short Scale‐3 (GSE‐3), Big Five Inventory Scale (BFI), Revised Life Orientation Test (LOT‐R) and Pain Catastrophizing Scale (PCS). Psychological readiness to return to sport was assessed with the ACJ‐Return to Sport after Injury (ACJ‐RSI) scale.

**Results:**

A total of 142 patients (mean age at surgery: 39.2 ± 13.7 years; 93 acute and 49 chronic injuries) with a mean follow‐up of 6.0 ± 2.2 years were included in the study. Patients had a mean CM of 85.2 ± 13.1, ASES of 93.0 ± 13.1, NC of 86.0 ± 13.8, VAS for pain of 2.0 ± 1.4 and ACJ‐RSI of 75.0 ± 23.7 at final follow‐up. The CM, ASES and NC scores were negatively correlated with the TSK (*ρ* = −0.30; *ρ* = −0.46; *ρ* = −0.35; *P* < 0.001, respectively) and the PCS (*ρ* = −0.32; *ρ* = −0.42; *ρ* = −0.30; *P* < 0.001, respectively), while for the GSE‐3 a positive correlation was observed (*ρ* = 0.32; *ρ* = 0.40; *ρ* = 0.42; *P* < 0.001, respectively). LOT‐R for optimism was positively correlated with ASES (*ρ* = 0.29; *P* = 0.001) and NC (*ρ* = 0.27; *P* = 0.003), while LOT‐R for pessimism was only negatively correlated with ASES (*ρ* = −0.20; *P* = 0.043). Regarding the BFI, neuroticism was negatively correlated with ASES (*ρ* = −0.24; *P* = 0.008), CM (*ρ* = −0.22; *P* = 0.017) and NC (*ρ* = −0.23; *P* = 0.013).

**Conclusion:**

Patient‐specific psychological characteristics and personality structure significantly affect functional outcomes after arthroscopically assisted ACJ stabilization for acute and chronic ACJ injuries at mid‐term follow‐up. Functional outcome scores were positively correlated with the patients' self‐efficacy and optimistic outlook on life, while for kinesiophobia, catastrophizing of pain and neuroticism, a negative correlation was observed.

**Level of Evidence:**

Level IV retrospective study.

**Abbreviations**
AC: acromioclavicular
ACJ: acromioclavicular joint
ACJ‐RSI: ACJ‐return to sport after injury
ASES: American Shoulder and Elbow Surgeons
BFI: Big Five Inventory
CC: coracoclavicular
CM: Constant–Murley
GSE‐3: General Self‐Efficacy Short Scale‐3
HT: hamstring tendon
ISAKOS: International Society of Arthroscopy, Knee Surgery and Orthopaedic Sports Medicine
LOT‐R: Revised Life Orientation Test
NC: Nottingham Clavicle
PCS: Pain Catastrophizing Scale
ROM: range of motion
TSK: Tampa Scale of Kinesiophobia
VAS: Visual Analogue Scale

## INTRODUCTION

Acromioclavicular joint (ACJ) injuries represent up to 12% of traumatic shoulder injuries and typically show a high incidence in the young and active population [4, 20, 30]. Contemporary evidence still supports surgical stabilization of higher‐grade ACJ injuries in specific cases, with the technique being used depending on the chronicity of injury [3, 11, 23, 33]. Accordingly, biologic augmentation using a tendon auto‐ or allograft should be performed in chronic ACJ injuries (>3 weeks after injury), which has been implemented in current treatment guidelines [4, 33].

Although a growing body of evidence reports favourable and reliable postoperative outcomes following arthroscopically assisted ACJ stabilization both in the acute and chronic setting [18, 23, 36, 38], there is currently no evidence pertaining to the influence of patient‐specific psychological characteristics and personality structure on outcomes after surgical stabilization of ACJ injuries. Evaluating the impact of psychological factors is of great clinical importance, as in patients reporting unsatisfactory postoperative outcomes, the underlying reasons are not always clearly attributable to objective clinical or radiological findings.

Consequently, other factors may significantly influence postoperative outcomes in these patients. Recently, the patients' psychological characteristics and personality structure have gained increasing importance in explaining both favourable and unfavourable postoperative results after knee or shoulder surgery [12, 13, 15, 17, 21, 27, 34, 37, 42]. More specifically, common key factors affecting postoperative satisfaction, functional outcomes and return to sports include fear of movement (kinesiophobia), catastrophizing of pain, the patient's belief in their own ability to manage challenging life situations (self‐efficacy) as well as the extent of either optimistic or pessimistic outlook on life [12, 13].

Thus, the purpose of the study was to evaluate the impact of patient‐specific psychological factors and personality structure on functional outcomes of patients undergoing isolated ACJ stabilization for acute and chronic ACJ injuries. It was hypothesized that patient‐specific psychological factors and personality structure would significantly affect functional outcomes after ACJ stabilization for acute and chronic ACJ injuries at mid‐term follow‐up.

## METHODS

This was an Institutional‐Review‐Board (2022‐223‐S‐Np) approved retrospective monocentric outcome study. An institutional data bank review was performed to identify patients who underwent ACJ stabilization for isolated acute or chronic Type III–V ACJ injuries (according to the Rockwood classification [32]) between January 2015 and December 2021 and had a minimum follow‐up of 2 years. Patients were excluded, if the ACJ injury was treated conservatively or was not classified as Rockwood Type III–V in the retrospective radiographic review. Further, patients undergoing concomitant surgical procedures (e.g., long head of the biceps tenodesis/tenotomy, rotator cuff repair, labral repair) were excluded. Informed consent was obtained from each patient.

### Surgical technique

All patients were treated with the same surgical technique based on a suspensory fixation. Arthroscopically assisted coracoclavicular (CC) and acromioclavicular (AC) stabilization was performed as previously described [5]. The patient was placed in the beach‐chair position. A posterior and lateral viewing portal, as well as a low anterior working portal, were established. The coracoid base was dissected using an electrothermal ablation device. An incision was made over the AC joint. Articular disc tissue preventing reduction was removed. Tunnels in the lateral clavicle and acromion were drilled 10 mm parallel to the ACJ line using a 2.4‐mm cannulated drill. A high‐strength suture tape (FiberTape; Arthrex Inc.) was passed through the drill holes. Next, the ACJ was reduced under fluoroscopy, and temporary K‐wire fixation was performed. Through a second incision over the lateral clavicle (3.5 cm medial to the AC joint line), a tunnel was drilled through the clavicle and coracoid using a 2.4‐mm drill. Two suture tapes (FiberTape) were passed through the drill holes and threaded through two titanium buttons (DogBone; Arthrex Inc.). Finally, both the AC and CC constructs were tightened and knotted for AC and CC stabilization. In the chronic situation, the CC and AC construct was augmented with a gracilis tendon autograft, which was shuttled along with the suture tape construct, and recovered from superiorly anterior to the clavicle. The ends of the gracilis autograft were then tied, and the knot was reinforced with multiple stitches of an absorbable suture. For graft passage, a 4‐mm drill was used [5].

### Postoperative rehabilitation

The operated arm was immobilized in a sling for 6 weeks. Limited active‐assisted range‐of‐motion exercises were gradually increased over 6 weeks. Active full range‐of‐motion exercises were started after 6 weeks, followed by a gradual return to overhead activities with load after 12 weeks, after clinical and radiographic control. Patients were advised to wait for 6 months before returning to full‐contact sports. Return to work was coordinated in accordance with the return‐to‐sports protocol. Rehabilitation protocols did not differ based on aetiology.

### Functional outcome parameters

Functional outcome measures included the Constant–Murley (CM), American Shoulder and Elbow Surgeons (ASES) and Nottingham Clavicle (NC) score as well as the Visual Analogue Scale (VAS) for pain, which were assessed at final follow‐up.

### Psychological characteristics and personality structure

Patient‐specific psychological characteristics and personality structure were evaluated using the Tampa Scale of Kinesiophobia (TSK‐11), Pain Catastrophizing Scale (PCS), General Self‐Efficacy Short Scale‐3 (GSE‐3), Big Five Inventory Scale (BFI) and Revised Life Orientation Test (LOT‐R), which were collected at final follow‐up.

To measure the fear of movement, the short form of the Tampa Scale of Kinesiophobia (TSK‐11) was utilized in the German language. The objective of this score is to assess pain‐related coping behaviours and kinesiophobia based on eleven statements, with a higher total score indicating a greater level of kinesiophobia [9, 29].

To assess pain catastrophizing, the PCS was applied. The experience of feelings and thoughts related to pain is assessed based on 13 statements. Respondents indicate on a scale the extent to which these statements reflect their sensations. A higher total score indicates a greater level of catastrophizing [22].

To assess the patients' sense of self‐efficacy, the GSE‐3 was used. In this context, general self‐efficacy refers to the confidence in one's ability to master difficult life situations or health‐related challenges through personal competencies. This score is standardized with three questions to assess this concept [2, 16].

Additionally, the BFI was queried. The ‘Big Five’ describe the five dimensions of personality, categorized based on the five‐factor model as extraversion, neuroticism, openness, conscientiousness and agreeableness. The BFI is an economical short scale consisting of ten questions designed to measure and assign these dimensions. Research has demonstrated that individuals with varying personality traits exhibit differences in health behaviours and experiences of stress [31, 40, 43].

The German version of the LOT‐R was implemented to assess the general optimism or pessimism of patients [14]. Numerous studies have demonstrated that an optimistic attitude is associated with better physical and psychological well‐being, health behaviours and recovery outcomes [39, 44].

### Readiness to return to sport

Psychological readiness to return to sport was assessed with the ACJ‐Return to Sport after Injury (ACJ‐RSI) scale. This scale includes 12 items that assess risk perception, confidence in the ACJ and emotions. The ACJ‐RSI scale was adapted from the previously published RSI scales for shoulder instability and anterior cruciate ligament reconstruction [28, 41].

### Statistical analysis

An a priori power analysis was performed based on pilot data and Cohen's conventions. In the pilot data, the strongest correlation with the ASES score was observed for the TSK with an effect size of *r* = 0.45. However, this estimate was considered unrealistically high, as it would have resulted in a required sample size of only 33 patients to reliably detect this effect with a power of 80% at an *α*‐level of 0.05. Consequently, for the a priori sample size calculation, it was deliberately chosen not to base the analysis on the strongest observed effect but instead to use a more conservative approach based on Cohen's conventions [7]. According to Cohen's convention, an *r* = 0.30 represents a medium effect size, meaning that the psychological predictors are assumed to explain a moderate proportion of the variance in the ASES score. Assuming a medium effect size of *r* = 0.30 according to Cohen's conventions, with a significance level of *α* = 0.05 (two‐tailed) and a statistical power of 80%, a sample size of 82 patients is required. The power analysis was performed with G*Power (Erdfelder, Faul, Buchner, Lang; HHU Düsseldorf) [10].

Descriptive statistics were used to summarize categorical and continuous variables, with categorical variables reported as counts and percentages, and continuous variables reported as mean ± standard deviation. The Shapiro–Wilk test was used to evaluate the distribution of continuous variables. Parametric tests (unpaired *t* test) or non‐parametric tests (Mann–Whitney *U* test) were used to compare continuous variables between groups, depending on the respective distribution of the data. Categorical variables were compared using the binary Fisher's exact test or *χ* ^2^ test as appropriate. Correlations between psychological and functional scores were assessed using the Pearson or Spearman correlation coefficient, where appropriate. A subgroup analysis was performed for acute and chronic ACJ injuries. Confidence intervals of 95% were calculated, and a significance level of *P* < 0.05 was used. The statistical analysis was performed using SPSS software version 29.0 (IBM‐SPSS).

## RESULTS

**Figure 1:**
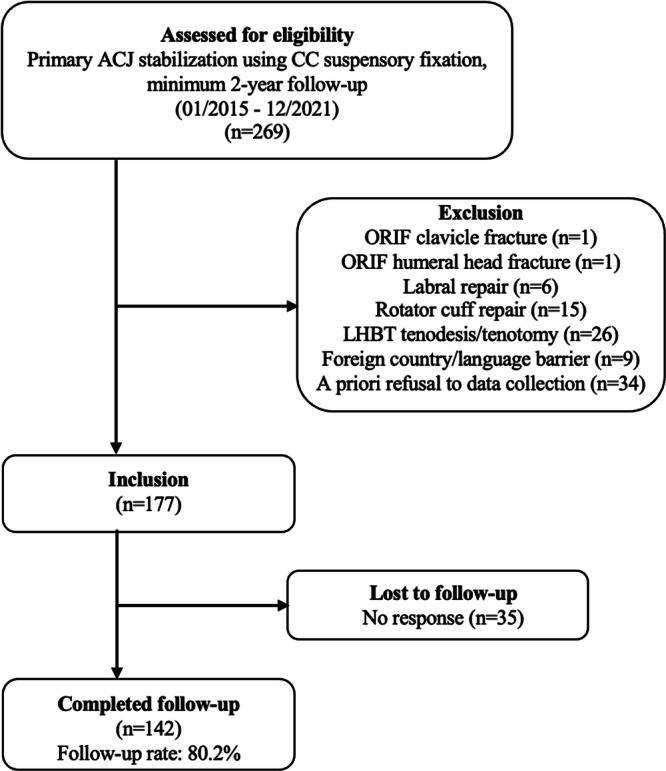
Flowchart visualizing the patient population of the acute and chronic group after accounting for inclusion and exclusion criteria as well as those lost to follow‐up. AC, acromioclavicular; ACJ, acromioclavicular joint; CC, coracoclavicular; LHBT, long head of the biceps tendon; ORIF, open reduction internal fixation.

A total of 269 patients were assessed for eligibility following a review of the institutional database between January 2015 and December 2021. Of those, a total of 142 patients (mean age at surgery: 39.2 ± 13.7 years; 93 acute and 49 chronic injuries) with a mean follow‐up of 6.0 ± 2.2 years (range: 2.0–11.0 years) were included in the final analysis. A flowchart detailing the inclusion process is provided in Figure 1. The follow‐up time in the acute and chronic groups was similar (*P* = 0.244), while patients in the chronic group were significantly older (*P* = 0.009) and showed a longer time duration from injury to surgery (*P* < 0.001). Demographic characteristics of the patient population are provided in Table 1. Six patients in the acute group and three patients in the chronic group failed and underwent revision surgery (acute: 6.5%; chronic: 6.1%; *P* = 0.94).

**Table 1:** Demographic characteristics of the subject population.

	Total (*N* = 142)	Acute injuries (*N* = 93)	Chronic injuries (*N* = 49)
Age at surgery (years)	39.2 ± 13.7	37.0 ± 13.1	43.4 ± 14.0
Follow‐up time (years)	6.0 ± 2.2	6.1 ± 2.0	5.8 ± 2.6
Time from injury to surgery (days)	155.2 ± 426.6	12.4 ± 11.4	426.2 ± 647.9
Sex			
Male (%)	121 (85.2)	85 (91.4)	36 (73.5)
Female (%)	21 (14.8)	8 (8.6)	13 (26.5)
Rockwood type			
III (%)	29 (20.4)	29 (31.2)	
IIIb (%)	20 (14.1)		20 (40.8)
V (%)	93 (65.5)	64 (68.8)	29 (59.2)
Surgical technique			
CC	19 (13.4)	19 (20.4)	0 (0.0)
CC + AC	123 (86.6)	74 (79.6)	49 (100.0)

Abbreviations: AC, acromioclavicular; CC, coracoclavicular.

### Functional outcomes

At final follow‐up, patients had a mean CM of 85.2 ± 13.1, ASES of 93.0 ± 13.1, NC of 86.0 ± 13.8 and VAS for pain of 2.0 ± 1.4 (Table 2). There was no significant difference in outcome scores between acute and chronic ACJ injuries, Type III/IIIb and V ACJ injuries as well as male and female patients (*P* > 0.05, respectively).

**Table 2:** Functional outcome measures.

Outcome measure	Total	Acute	Chronic	*p* value^a^
ASES	93.0 ± 13.1	94.5 ± 9.7	90.2 ± 17.6	0.470
Constant–Murley	85.2 ± 13.1	86.5 ± 12.3	82.8 ± 14.4	0.134
Nottingham Clavicle	86.0 ± 13.8	87.2 ± 12.4	83.7 ± 16.1	0.337
VAS Pain	2.0 ± 1.4	1.9 ± 1.1	2.3 ± 1.8	0.546

Abbreviations: ASES, American Shoulder and Elbow Surgeons; VAS, Visual Analogue Scale.

a Comparison between acute and chronic injuries.

### Psychological characteristics and personality structure

Regarding the patient‐specific psychological characteristics and personality structure, a TSK of 17.7 ± 5.6, GSE‐3 of 13.8 ± 1.5 and PCS of 5.2 ± 8.3 as well as a LOT‐R for optimism of 11.6 ± 2.2 and LOT‐R for pessimism of 6.9 ± 2.3 were observed (Table 3). For the five dimensions of the BFI, patients showed an extraversion of 3.6 ± 0.9, neuroticism 2.4 ± 0.9, openness 3.5 ± 0.9, conscientiousness 4.0 ± 0.7 and agreeableness 3.3 ± 0.8. There was no significant difference in psychological characteristics and personality structure between acute and chronic ACJ injuries or type III/IIIb and V ACJ injuries (*P* > 0.05, respectively).

**Table 3:** Psychological characteristics and personality structure.

Outcome measure	Total	Acute	Chronic	*p* value^a^
TSK	17.7 ± 5.6	17.6 ± 5.3	17.7 ± 6.2	0.727
GSE‐3	13.8 ± 1.5	13.9 ± 1.4	13.6 ± 1.7	0.511
PCS	5.2 ± 8.3	4.1 ± 6.8	7.2 ± 10.5	0.239
LOT‐R optimism	11.6 ± 2.2	11.4 ± 2.2	12.0 ± 2.1	0.230
LOT‐R pessimism	6.9 ± 2.3	6.8 ± 2.4	7.0 ± 2.2	0.647
BFI				
Extraversion	3.6 ± 0.9	3.6 ± 0.8	3.6 ± 0.9	0.720
Neuroticism	2.4 ± 0.9	2.4 ± 0.8	2.4 ± 0.9	0.739
Openness	3.5 ± 0.9	3.5 ± 0.9	3.4 ± 0.9	0.283
Conscientiousness	4.0 ± 0.7	4.0 ± 0.7	4.1 ± 0.7	0.299
Agreeableness	3.3 ± 0.8	3.3 ± 0.8	3.3 ± 0.8	0.837

Abbreviations: BFI, Big Five Inventory Scale; GSE‐3, General Self‐Efficacy Short Scale‐3; LOT‐R, Revised Life Orientation Test; PCS, Pain Catastrophizing Scale; TSK, Tampa Scale of Kinesiophobia.

a Comparison between acute and chronic injuries.

### Correlation of functional outcomes with psychological characteristics

The CM, ASES and NC scores were negatively correlated with the TSK (*ρ* = −0.30; *ρ* = −0.46; *ρ* = −0.35; *P* < 0.001, respectively) and the PCS (*ρ* = −0.32; *ρ* = −0.42; *ρ* = −0.30; *P* < 0.001, respectively), while for the GSE‐3 a positive correlation was observed (*ρ* = 0.32; *ρ* = 0.40; *ρ* = 0.42; *P* < 0.001, respectively). LOT‐R for optimism was positively correlated with ASES (*ρ* = 0.29; *P* = 0.001) and NC (*ρ* = 0.27; *P* = 0.003), while LOT‐R for pessimism was only negatively correlated with ASES (*ρ* = −0.20; *P* = 0.043) (Table 4). When looking at the BFI, only the dimension neuroticism was negatively correlated with the ASES (*ρ* = −0.24; *P* = 0.008), CM (*ρ* = −0.22; *P* = 0.017) and NC (*ρ* = −0.23; *P* = 0.013), while all other dimensions showed no significant correlation to functional outcome scores. The correlations between psychological characteristics and functional outcome scores were similar for both acute and chronic ACJ injuries.

**Table 4:** Correlation of functional outcomes with psychological characteristics.

	CM			ASES			NC		
	*ρ*	*p* value	95% CI	*ρ*	*p* value	95% CI	*ρ*	*p* value	95% CI
TSK	−0.30	<0.001^a^	−0.46 to −0.12	−0.46	<0.001^a^	−0.59 to −0.30	−0.35	<0.001^a^	−0.50 to −0.17
GSE‐3	0.32	<0.001^a^	0.14 to 0.47	0.40	<0.001^a^	0.24 to 0.55	0.42	<0.001^a^	0.26 to 0.56
PCS	−0.32	<0.001^a^	−0.48 to −0.15	−0.42	<0.001^a^	−0.56 to −0.26	−0.30	<0.001^a^	−0.46 to −0.13
LOT‐R optimism	0.11	0.228	−0.08 to 0.29	0.29	0.001^a^	0.11 to 0.45	0.27	0.003^a^	0.09 to 0.43
LOT‐R pessimism	−0.02	0.846	−0.20 to 0.17	−0.19	0.043^a^	−0.36 to −0.01	−0.16	0.083	−0.34 to 0.03
BFI									
Extraversion	0.11	0.218	−0.07 to 0.29	−0.09	0.323	−0.27 to 0.10	0.03	0.729	−0.15 to 0.22
Neuroticism	−0.22	0.017^a^	−0.39 to −0.04	−0.24	0.008^a^	−0.41 to −0.06	−0.23	0.013^a^	−0.40 to −0.04
Openness	0.05	0.585	−0.14 to 0.23	0.02	0.982	−0.18 to 0.19	−0.03	0.721	−0.22 to 0.15
Conscientiousness	−0.05	0.581	−0.23 to 0.14	0.08	0.402	−0.11 to 0.26	0.13	0.149	−0.05 to 0.31
Agreeableness	0.07	0.483	−0.12 to 0.25	0.14	0.136	−0.05 to 0.32	−0.06	0.547	−0.24 to 0.13

Abbreviations: ASES, American Shoulder and Elbow Surgeons Score; BFI, Big Five Inventory Scale; CI, confidence interval; CM, Constant–Murley Score; GSE‐3, General Self‐Efficacy Short Scale‐3; LOT‐R, Revised Life Orientation Test; NC, Nottingham Clavicle Score; PCS, Pain Catastrophizing Scale; TSK, Tampa Scale of Kinesiophobia.

a Statistically significant correlations.

### Readiness to return to sport

Overall, patients had an ACJ‐RSI of 75.0 ± 23.7, with no significant difference between acute (74.4 ± 23.8) and chronic (76.1 ± 23.7) ACJ injuries (*P* = 0.607). The ACJ‐RSI was significantly correlated with the CM (*ρ* = 0.42), ASES (*ρ* = 0.58) and NC (*ρ* = 0.64) as well as with the TSK (*ρ* = −0.46), GSE‐3 (*ρ* = 0.32), PCS (*ρ* = −0.40) and LOT‐R for optimism (*ρ* = 0.35) (Table 5). The correlations of psychological characteristics and functional outcome scores with the ACJ‐RSI were similar for both acute and chronic ACJ injuries.

**Table 5:** Correlation of functional outcomes and psychological characteristics with ACJ‐RSI.

	ACJ‐RSI		
	*ρ*	*p* value	95% CI
CM	0.42	<0.001^a^	0.25–0.56
ASES	0.58	<0.001^a^	0.44–0.69
NC	0.64	<0.001^a^	0.51–0.74
TSK	−0.46	<0.001^a^	−0.60 to −0.30
GSE‐3	0.32	<0.001^a^	0.14–0.48
PCS	−0.40	<0.001^a^	−0.55 to −0.23
LOT‐R optimism	0.35	<0.001^a^	0.17–0.50
LOT‐R pessimism	−0.12	0.193	−0.30 to 0.07
BFI			
Extraversion	0.01	0.985	−0.18 to 0.19
Neuroticism	−0.10	0.284	−0.28 to 0.09
Openness	−0.01	0.926	−0.19 to 0.18
Conscientiousness	0.13	0.167	−0.06 to 0.31
Agreeableness	0.07	0.475	−0.12 to 0.25

Abbreviations: ACJ‐RSI, Acromioclavicular Joint—Return to Sport after Injury Scale; ASES, American Shoulder and Elbow Surgeons Score; BFI, Big Five Inventory Scale; CI, confidence interval; CM, Constant–Murley Score; GSE‐3, General Self‐Efficacy Short Scale‐3; LOT‐R, Revised Life Orientation Test; NC, Nottingham Clavicle Score; PCS, Pain Catastrophizing Scale; TSK, Tampa Scale of Kinesiophobia.

a Statistically significant correlations.

## DISCUSSION

The most important finding of the study was that patient‐specific psychological characteristics and personality structure significantly affect functional outcomes after arthroscopically assisted ACJ stabilization for acute and chronic ACJ injuries at mid‐term follow‐up. In addition, there was no significant difference in outcome scores and failure rates between acute and chronic ACJ injuries. These data are helpful for shoulder surgeons when managing patients' expectations regarding postoperative functional success after ACJ stabilization in the acute or chronic setting based on the patient's specific psychological characteristics and personality structure. Further, the consideration of psychological factors may represent another key component in the decision‐making process regarding the indication for surgical intervention in patients with ACJ injuries.

Current evidence still supports a surgical stabilization of higher‐grade ACJ injuries depending on the specific functional and cosmetic demands of the patient [3, 11, 23, 24, 33]. Available literature shows favourable and reliable postoperative outcomes following arthroscopically assisted ACJ stabilization [18, 23, 36, 38], with the previously reported postoperative functional outcomes scores and failure rates being consistent with the present study comprising a very large subject population of 142 patients. However, not all patients may benefit from ACJ stabilization to the expected and desired extent.

Interestingly, it has been observed that in some patients reporting unsatisfactory postoperative outcomes after shoulder surgery, the underlying reasons are not always clearly attributable to objective clinical or radiological findings [12, 13]. Recently, patient‐specific psychological characteristics and personality structure have gained increasing importance in explaining both favourable and unfavourable postoperative results after knee or shoulder surgery [12, 13, 15, 17, 21, 27, 34, 37, 42]. This particularly includes kinesiophobia, catastrophizing of pain, the patient's self‐efficacy as well as the extent of either optimistic or pessimistic outlook on life [12, 13].

Evidence pertaining to the influence of patient‐specific psychological characteristics and personality structure on outcomes after surgical stabilization of ACJ injuries is highly limited. Of note, the data of the present study demonstrates that functional outcome scores after ACJ stabilization in the acute or chronic setting were positively correlated with the patients' self‐efficacy and optimistic outlook on life at mid‐term follow‐up, while for kinesiophobia, catastrophizing of pain and neuroticism, a negative correlation was observed. This indicates that patients with a greater belief in their own ability to handle difficult or challenging life situations (self‐efficacy) and a more optimistic outlook on life achieve better functional outcomes postoperatively. In contrast, patients with a greater fear of movement (kinesiophobia), increased catastrophic thinking when experiencing pain and a pronounced personality trait in the dimension of neuroticism reported poorer functional outcomes. More specifically, characteristics typically associated with neuroticism include a tendency to nervosity, sadness and uncertainty, irritability as well as complaints of anxiety and physical pain. In addition, readiness to return to sport was positively correlated with self‐efficacy and optimism, while there was a negative correlation with kinesiophobia and pain catastrophizing. These observations are also helpful when managing patients' expectations regarding postoperative functional success after ACJ stabilization. In patients scheduled for surgical stabilization showing greater self‐efficacy and a more optimistic outlook on life, postoperative success is more likely to be achieved compared to those with lower self‐efficacy, a more pessimistic outlook on life, kinesiophobia, pain catastrophizing or and a pronounced personality trait in the dimension of neuroticism. This should be an integral part of the decision‐making process and patient education to assess if a patient should be indicated for ACJ stabilization in the first place.

Against the background of a constantly increasing number of patients with successful conservative treatment of higher‐grade ACJ injuries [1, 25, 35], it is especially important to consider ACJ stabilization only in those patients who could really benefit from surgical intervention without potential impairment of postoperative results due to disadvantageous psychological characteristics or personality traits. Consequently, the present data may help to screen for patients with expectable worse postoperative outcomes based on their personality structure, which may make them not ideal candidates for undergoing surgical ACJ stabilization. Conversely, if a patient with kinesiophobia or pain catastrophizing undergoes surgery, more support during the postoperative rehabilitation may be required. Practically, such a screening can be performed during the patient's visit. Prior to discussing the treatment options, the patient's psychological scores can be collected to gain an impression of the patient's personality structure. Patients showing personality traits associated with poor functional outcomes (kinesiophobia, pain catastrophizing, pessimism, neuroticism, etc.) may be better suited for conservative treatment.

As a secondary observation, the present study showed no significant differences in functional outcomes between patients who underwent surgery either in the acute or chronic setting. Previous evidence pertaining to the impact of chronicity on outcomes following ACJ is limited [8, 19]. Existing comparative studies only have small sample sizes while showing inconsistent and rather conflictive results [8, 19]. More specifically, Dey Hazra et al. found a statistically significant superiority of early surgical stabilization for type IIIb and V ACJ injuries compared to delayed surgery (>3 weeks) in terms of functional scores at a mean follow‐up of 3.2 years [8]. On the contrary, Lädermann et al. observed that early and delayed surgical intervention resulted in equivalent clinical outcomes for Type III–V ACJ injuries at a mean follow‐up of 3.5 years, which is consistent with the data of the present study, where both acute and chronic injuries achieved similar postoperative functional outcomes [19]. Although delayed surgery requires augmentation using a tendon graft, the equality of delayed to early intervention is important information when counselling patients regarding the urgency of surgery.

The study has several limitations. The study inherits the associated biases of a retrospective design. As no baseline values of the psychological and functional scores were available, the true improvement of each patient from pre‐ to postoperatively remains unknown. Further, this study cannot capture a potential change of psychological characteristics or personality traits triggered by the event of an ACJ dislocation or during the postoperative course. Second, the monocentric study design may limit the external validity of the results as surgery was performed in a single reference centre for arthroscopic stabilization of ACJ injuries. Third, the ACJ‐RSI was adapted from the previously published RSI scales for shoulder instability and anterior cruciate ligament reconstruction without prior validation. Finally, as the correlation of functional outcomes with psychological characteristics was elected as the primary endpoint of this study, a postoperative radiological evaluation at final follow‐up was not conducted. The decision was made not to expose patients to additional radiation, as previous studies did not reveal an association between radiological reduction and clinical findings after ACJ stabilization [6, 26]. Consequently, loss of radiographic reduction as well as the potential presence of iatrogenic fractures, tunnel widening, heterotopic ossifications, osteoarthritis or osteolysis around the ACJ could not be evaluated.

## CONCLUSION

Patient‐specific psychological characteristics and personality structure significantly affect functional outcomes after arthroscopically assisted ACJ stabilization for acute and chronic ACJ injuries at mid‐term follow‐up. Functional outcome scores were positively correlated with the patients' self‐efficacy and optimistic outlook on life, while for kinesiophobia, catastrophizing of pain and neuroticism, a negative correlation was observed.

## AUTHOR CONTRIBUTIONS

*Conceptualization*: Lukas Nawid Muench and Bastian Scheiderer. *Methodology*: Lukas Nawid Muench, Armin Runer, Bastian Scheiderer and Frank Martetschläger. *Formal analysis and investigation*: Lukas Nawid Muench, Ali Can Gunenc, Lisa Rahn and Marco‐Christopher Rupp. *Writing—original draft preparation*: Lukas Nawid Muench, Bastian Scheiderer and Marco‐Christopher Rupp. *Writing—review and editing*: Sebastian Siebenlist, Armin Runer, Lucca Lacheta and Frank Martetschläger. *Funding acquisition*: No funding. *Resources*: Sebastian Siebenlist and Bastian Scheiderer. *Supervision*: Lucca Lacheta, Bastian Scheiderer, Frank Martetschläger and Sebastian Siebenlist.

## CONFLICT OF INTEREST STATEMENT

Bastian Scheiderer is a consultant for Arthrex GmbH. Sebastian Siebenlist is a consultant for Arthrex GmbH, KLS Martin Group and medi GmbH & Co. KG. Marco‐Christopher Rupp has received payments/honoraria and support for attending meetings/travel/fellowships from Arthrex GmbH. The remaining authors declare no conflict of interest.

## ETHICS STATEMENT

This retrospective chart review study involving human participants was in accordance with the ethical standards of the institutional and national research committee and with the 1964 Helsinki Declaration and its later amendments or comparable ethical standards. The Human Investigation Committee (IRB) of University B approved this study (IRB No. 2022‐223‐S‐Np). Informed consent was obtained from all individual participants included in the study.

## Data Availability

The authors have nothing to report.
